# Disposal Practice of Unfit Medicines in Nongovernmental Hospitals and Private Medicine Outlets Located in Mwanza, Tanzania

**DOI:** 10.1155/2019/7074959

**Published:** 2019-03-03

**Authors:** Stanley Mwita, Godfrey Ngonela, Deogratias Katabalo

**Affiliations:** ^1^School of Pharmacy, Catholic University of Health and Allied Sciences (CUHAS), P.O. Box 1464, Mwanza, Tanzania; ^2^Shinyanga Regional Referral Hospital, P.O. Box 17, Shinyanga, Tanzania

## Abstract

**Introduction:**

For a medicine to qualify as safe and effective and to be of good quality, it should be properly labelled, stored, and transported. If a medicine is not handled properly, it ends up being unfit. Improper disposal of unfit medicines contributes to the appearance of their metabolites in the environment.

**Methods:**

A descriptive cross-sectional study was designed to capture quantitative data. The study was conducted in Mwanza region, Tanzania. The study population comprised nongovernmental hospitals and private medicines outlets in the region. The sample size was 111 facilities. This study was conducted between October 2013 and May 2014. The questionnaire was used to assess experience and challenges of dealing with unfit medicines. A review of waste management records was done to capture data of past disposal for unfit medicines. The coded data were analyzed using Statistical Package for Social Sciences (Version 20.0) computer analysis software. Comparison of proportions between groups was performed using Pearson's chi square.

**Results:**

The majority of facilities (41.4%) used methods such as the pouring of unfit medicines into the sink and into the dustbin. About 60.4% of facilities were found with unfit medicines at the time of survey. Majority of unfit medicines found were antibiotics (64.1%). Almost 10% of health facilities maintained a register book for recording unfit medicines.

**Conclusion:**

There was improper disposal of unfit medicines in health facilities studied, whereby commonly reported methods of disposal were pouring into the sink and putting into the street dustbin. In private medicines outlets, there was poor storage management practice as some of the unfit medicines were left unpacked into boxes or separated from the usable medicines and not properly labelled.

## 1. Introduction

Medicines are commonly used by the healthcare system to diagnose, treat, or prevent illnesses. Because of their importance in improving public health services, regulatory processes concerning their quality are necessary to ensure that intended treatment outcomes are met. For a medicine to qualify as safe and effective and to be of good quality, it should be properly labelled, stored, and transported. If a medicine is not handled properly, it ends up being unfit. Healthcare activities generate significant amounts of medical wastes such as unfit medicines. The management of medical waste is an integral part of a national healthcare system [1].

According to the Tanzania Food and Drugs Authority (TFDA) Guidelines for safe disposal of unfit medicines and cosmetic products, medicines are considered unfit when they are expired, improperly sealed, damaged, improperly stored, improperly labelled, counterfeit, substandard, adulterated, prohibited, and unauthorized [2].

Improper disposal of unfit medicines contributes to the appearance of their metabolites in the environment [3]. Traces of pharmaceutical residues have consistently been detected in effluents from sewerage facilities, surface water, and drinking water [4–7]. The rate of medical waste generation is high, about 2,250 kg/day to 2,500 kg/day [8]. Controlled high-temperature incineration is the appropriate disposal method process rather than open pit burning or burying [9, 10]; however, the main disposal methods for unfit medicines in health facilities (HF) comprises open pit burning (48%), burying (29%), and incomplete low-temperature incineration (19%) [11, 12]. Open pit burning and uncontrolled incomplete incineration emit toxic air pollutants and toxic ash residues that are the major source of dioxins in the environment [13]. During incineration, if no proper filtering of flue gases is done, air can be polluted causing airborne illnesses to the nearby populations [14]. Dumping of healthcare waste in the uncontrolled areas can have a direct environmental effect by contaminating soils and underground water [15].

The absence of proper management of medical wastes such as unfit medicines and poor control of their disposal is the most critical problems connected with medical wastes [16]. In many countries, medical wastes are still handled and disposed off together with domestic wastes, posing a great health risk to municipal workers, the public, and the environment [17, 18]. Medical waste must be separated from municipal waste [19, 20]. Results from a survey of hospitals in Metropolitan Lagos, Nigeria, to assess medical waste management (MWM) practices and their implications to health and environments revealed a high rate of noncompliance to the Nigerian National Guidelines for MWM, whereby medical waste, including medicines from the surveyed facilities, was disposed off alongside municipal solid waste. The main reason cited was weak regulatory enforcement by the relevant authorities [21].

Studies conducted in Tanzania regarding medical wastes had described MWM in Tanzania as being poor and that the general awareness on issues related to MWM is lacking among the medical waste generators (e.g., health facilities) and handlers (e.g., staff involved in handling wastes) [12]. Due to poor control of wastes, hospital owners do not inspect well how they handle and dispose off the wastes they produce; as a result, hazardous wastes reach the dumpsite without notice [22]. It is estimated that 10–25% of healthcare waste is hazardous [23]. This study intends to assess practices and challenges encountered in managing disposal of unfit medicines in nongovernmental hospitals and private medicines outlets in the Mwanza region. Such assessments endeavour to provide valuable information for local health policy makers, TFDA, health services providers in private sectors, and all other stakeholders involved in disposal of unfit medicines to effectively plan, manage, and supervise their safe disposal.

## 2. Methods and Materials

### 2.1. Study Design

A descriptive cross-sectional study was designed to capture quantitative data.

### 2.2. Study Sites

The study was conducted in the Mwanza region. Mwanza region is among 26 regions in the Tanzania mainland. The entire region has six districts, namely, Nyamagana, Ilemela, Kwimba, Misungwi, Magu, and Ukerewe.

### 2.3. Sample Size

All five nongovernmental hospitals were included. There were 56 pharmacies in Mwanza region (22 wholesale and 34 retail pharmacies); however, two pharmacies were not willing to participate, so 54 pharmacies were included. For Accredited Drug Dispensing Outlets (ADDO) shops, the sample size was 52, which were calculated using Yamane Taro, 1967. Thus, the study involved 111 health facilities.

### 2.4. Data Collection

The study was performed using records of all nongovernmental hospitals and private medicines outlet, and the participants were personnel involved directly with medicines. This study was conducted between October 2013 and May 2014. Data were collected by the use of a structured questionnaire (Annex I in Supplementary Materials). The questionnaire was used to assess experience and challenges on dealing with unfit pharmaceuticals. A review of records was done using a checklist (Annex II in Supplementary Materials) to capture data for the past disposal of unfit medicines. The record review collected information from the register book for recording unfit medicines, application form, and type of unfit medicines found in the health facility. This methodology helped to obtain actual information on the practices and procedures of safe disposal of unfit medicines. The method adopted for this study follows the procedure used by Longe and Williams [21].

### 2.5. Data Management and Analysis

All the collected data were counterchecked for their clarity and validity. The coded data were analyzed using Statistical Package for Social Sciences (Version 20.0) computer analysis software. Comparison of proportions between groups was performed using Pearson's chi square test or Fisher's exact test where appropriate. A *p* value of less than 0.05 was considered as statistically significant at 95% confidence interval.

### 2.6. Ethical Consideration

Ethical clearance was obtained from MUHAS Ethical Review Committee of Research and Publication. Permission to conduct study in the selected study sites was granted by owners of nongovernmental hospitals, pharmacy, and ADDO shops.

## 3. Results

### 3.1. Frequency of Disposal of Unfit Medicines

Responses with regard to the frequency of disposal of unfit medicines by the health facilities are summarized in Table 1.

The result shows that majority of facilities (83.8%) undertake the disposal of unfit medicines when necessary. Results show no difference between frequency of disposal with the type of facility (*p*=0.60).

### 3.2. Disposal Methods of Unfit Medicines

In order to ascertain disposal methods that are commonly used by the health facilities, respondents listed the following as the most common methods for unfit medicines disposal (Table 2).

Majority of facilities (41.4%) use methods like pouring of unfit medicines into the sink and putting into the dustbin. Results show no association between methods of disposal with the type of facility (*p*=0.42).

### 3.3. Storage Practices of Unfit Medicines Prior to Disposal

In order to ascertain the disposal practices of a particular health facility, the checklist was used to determine the procedures for handling unfit medicines prior to terminal disposal. The results of the checklist questions are shown in Table 3.

Only around 10% (10.8%) of health facilities surveyed maintained the register book for recording unfit medicines.

### 3.4. Type of Unfit Medicines Found in the Health Facility

About 60.4% of health facilities were found with unfit medicines at the time of survey, and majority of unfit medicines found were antibiotics (64.1%).

### 3.5. Barriers to Proper Disposal of Unfit Medicines

About 2% mentioned lack of areas for disposal as the barrier to proper disposal of unfit medicines.

About 40% of pharmacies mention long procedures for disposal of unfit medicines as the barrier.

## 4. Discussion

Medicines are vital for saving life in conditions of morbidity, but yet these medicines might become unfit before reaching the consumer. When medicines become unfit, they need more special care and handling than before and hence need special protocols to attend them. In most cases, authorities are failing to install appropriate systems for a variety of reasons, such as nonavailability of appropriate technologies, inadequate financial resources, and absence of professional training on waste management [24].

This study revealed that more than eighty percent of health facilities undertake disposal of unfit medicines when necessary, while almost ten percent dispose off unfit medicines annually (Table 1). This entails that many facilities have had no standard protocols to guide them on the timing and frequency with which disposal should be conducted. As a consequence, some of the facility owners decide to dispose off unfit medicines conveniently by pouring into sewerage system, burying at their homes, or burning in open spaces. Indeed, most of the health facilities do not have separate collection and disposal programmes for pharmaceutical waste [25].

The disposal methods most commonly used by nongovernmental hospitals and private medicine outlets were putting into the street dustbin or pouring into the sink (41.4%), (Table 2). No significant differences in disposal practice occurred between the types of health facility. A similar study in New Zealand revealed that 44.7% of private medicines outlets poured liquid medicines into the sink [26]. These practices increase potential danger for environmental pollution [27]. Improper disposal can contaminate the environment and pose significant risks to water, air, agricultural products, and food chain, even harm animals and livestock [4, 28]. Indeed, the majority of hospitals do not have incineration facility or access to other essential healthcare waste management equipment [6, 7]. Peer-reviewed literature on attitudes and practices to medicine disposal methods revealed that the most popular methods for medication disposal were in the garbage, toilet, or sink. Liquid medications were more likely to be rinsed down the sink, as opposed to solid tablets and capsules, which were more likely deposited in the rubbish bin [29].

The current study attempted to examine handling procedures of unfit medicines prior to terminal disposal. Most medicines' storekeepers and dispensers were interviewed on procedures prior to terminal disposal, and they mentioned the follows: isolation of unfit medicines from usable medicines, recording the medicines in a register, packing into boxes/keeping them in separate area, and labelling them. Results revealed poor storage management practices as some of the unfit medicines were left unpacked into boxes or separated from the usable medicines and not properly labelled, less than half of facilities are segregated unfit from usable medicines (Table 3).

**Figure 1:**
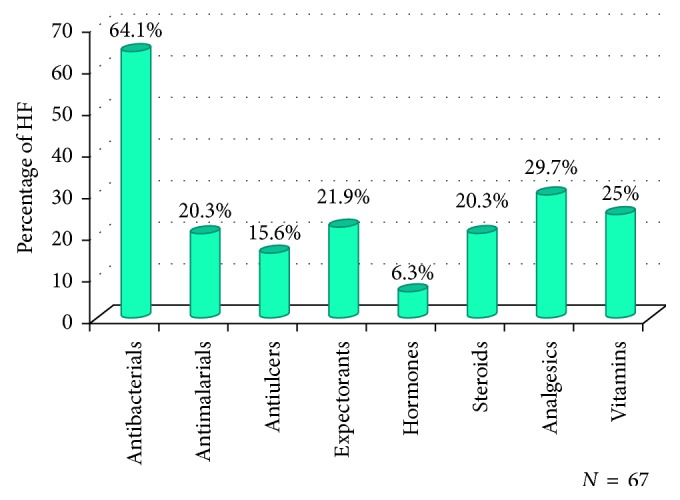
Types of unfit medicines found in the health facility.

About 60.4% of health facilities were found with unfit medicines at the time of survey, and more than half of unfit medicines were in the group of antibiotics (Figure 1). Evidence shows that the presence of antibiotics in water may lead to antibiotic resistance [5]. Studies on antibiotics have shown that up to 95% of antibiotic compounds can be released unaltered into the sewage system, and higher concentrations of antibiotics can lead to change in the microbial community structure and ultimately affect food chains [30]. Moreover, almost 30% of unfit medicines were analgesics (Figure 1). Nonsteroidal anti-inflammatory drugs (NSAIDs), like ibuprofen, naproxen, and aspirin, are the most commonly used drugs, which are usually found in effective quantities in municipal effluents [5, 31].

**Figure 2:**
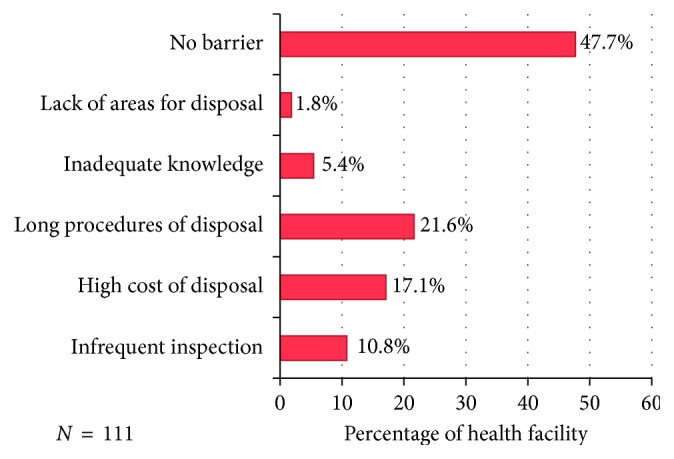
Barriers to proper disposal of unfit medicines.

**Figure 3:**
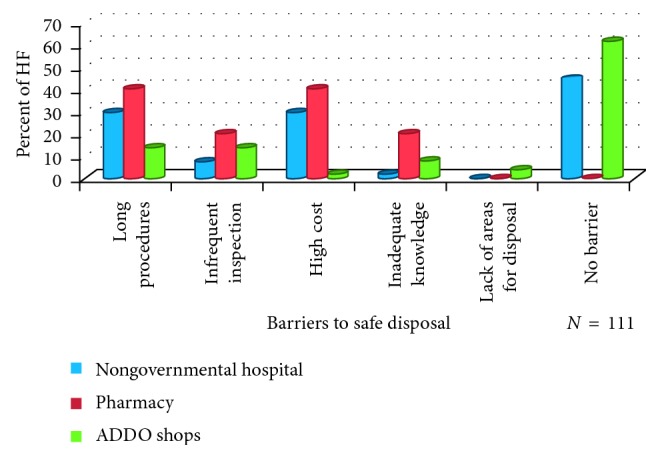
Barriers to proper disposal of unfit medicines according to health facility.

Almost half of health facilities (Figure 2) reported no barriers to disposal, and this might be due to the common practice used (pouring into the sink and putting into the street dustbin) does not need long procedures and extra resources. About 40% of pharmacies mentioned long procedures for disposal of unfit medicines as the barrier (Figure 3). The regulatory bodies need to do a better job at minimizing long disposal procedures and monitoring disposal practices for unfit medicines. Inadequacy in the MWM practices is mainly related to unsafe storage, lack of priority to the proper disposal, and poor control of waste disposal [32, 33]. Accumulation of unfit medicines poses three major concerns: (i) active pharmaceutical ingredients (APIs) disposed off into sewage or trash comprise a diverse source of potential chemical stressors in the environment. (ii) Accumulated medicines represent an increased potential for medicines diversion, with its attendant risks of unintentional poisonings and abuse. (iii) Leftover medicines represent wasted healthcare resources and lost opportunities for medical treatment [34]. The use of contractors in disposal of unfit medicines has shown to be the best way to minimize barriers in disposal of unfit medicines. Community pharmacists in New Zealand reported that 80.4% solid medications were removed by contractors [26]. However, this study was limited by the inability of the collected data to report on a detailed plethora of disposal methods which may have been used for the disposal of unfit medication rather than those commonly assumed by the authors.

Also, the research instrument was not able to collect, analyze, and report data on the percentage of participants who disposed off unfit medicines through the dustbin and sink separately. Further research to investigate other methods used apart from those reported in this study is a priority.

## 5. Conclusion

There was improper disposal of unfit medicines in health facilities studied whereby commonly reported methods of disposal were pouring into the sink and putting into the street dustbin. In private medicines outlets, there were poor storage management practices as some of the unfit medicines were left unpacked into boxes or separated from the usable medicines and not properly labelled. Medicines storekeepers and dispensers have a significant role in ensuring proper disposal and reducing the generation of unfit medicines. Doctors have a role for ensuring proper prescribing practices which do not result in an excess of unfit medicines. TFDA should enhance and strengthen its enforcement on nongovernmental hospitals and private medicine outlets through inspections and distribution of relevant guidelines. TFDA should register private companies in every region of the country on safe disposal of unfit medicines.

## Figures and Tables

**Table 1:** Frequency of disposal of unfit medicines.

Frequency of disposal	Type of HF							
	Nongovernmental hospital		Pharmacy		ADDO shops		Total	
	No.	%	No.	%	No.	%	No.	%
After six months	0	0	1	1.9	1	1.9	2	1.8
After every one year	0	0	9	16.7	5	9.6	14	12.6
After every 2 years	0	0	2	3.7	0	0	2	1.8
When necessary	5	100	42	77.8	46	88.5	93	83.8
Total	5	100	54	100	52	100	111	100

**Table 2:** Methods of disposal used by the health facility.

Methods of disposal used regularly	Type of HF							
	Nongovernmental hospital (*n*=5)		Pharmacy (*n*=54)		ADDO shops (*n*=52)		Total (*n*=111)	
	No.	%	No.	%	No.	%	No.	%
Landfill of untreated unfit medicines	2	40	8	14.8	13	25	23	20.7
Burning in open spaces	1	20	24	44.4	17	32.7	42	37.8
Others (pour into sink and put into dustbin)	2	40	22	40.7	22	42.3	46	41.4

**Table 3:** Storage practices of unfit medicines prior to disposal.

Storage practices of unfit medicines	Type of HF								*p*
	Nongovernmental hospitals (*n*=5)		Pharmacy (*n*=54)		ADDO shops (*n*=52)		Total (*n*=111)		
	No.	%	No.	%	No.	%	No.	%	
Maintained register	3	60	8	14.8	1	1.9	12	10.8	0.001
Segregated from usable medicines	4	80	28	51.9	13	25	45	40.5	0.002
Presence of separate area/box	4	80	14	25.9	4	7.7	22	19.8	<0.0001
Labelled properly	4	80	10	18.5	2	3.8	16	14.4	<0.0001

## Data Availability

The data used to support the findings of this study are available from the corresponding author upon request.

## Supplementary Materials

Supplementary Materials (i) Annex I: study questionnaire. (ii) Annex II: checklist on procedures of unfit medicines disposal.
