# Supplementary material for: Disposal Practice of Unfit Medicines in Nongovernmental Hospitals and Private Medicine Outlets Located in Mwanza, Tanzania
Source: J Environ Public Health. 2019 Mar 3;2019:7074959. doi: 10.1155/2019/7074959 (PMC6421723; doi:10.1155/2019/7074959)
Supplement: Supplementary Materials — (i) Annex I: study questionnaire. (ii) Annex II: checklist on procedures of unfit medicines disposal. [file 7074959.f1.pdf]

Annex I

Questionnaire: managing disposal of unfit pharmaceuticals by non-governmental hospitals and private medicine outlets in Mwanza -Tanzania.

Code No ………….. Date: …………………………

1. Name of the health facility……………………………………………………

2. Type

a) Private hospital

b) FBO hospital

c) Wholesale pharmacy

d) Retail pharmacy

e) ADDO shop

3. District…………………………………………………………

4. Does it happen that your medicines in the stock get expired/ damaged before use?

a) Yes

b) No

5. Explain how you handle such expired/ damaged medicines prior to terminal disposal ………………………………………………………………………………………………… ……………………………………………………….. …………………………………

6. How often do you dispose of unwanted stock of pharmaceuticals?

a) After six months

b) After every 1 years

c) After every 2 years

d) When necessary (no specified time period).

7. When was your last disposal of unfit products? Mention (month and year) ………………………………………………………………… (See verification form or any official document to verify).

8. What methods do you use regularly to dispose unfit medicines at your health facility?...
....

9. What do you think will be the dangers and problems associated with delay and improper disposal of unfit pharmaceuticals?

a) Relabeling and resold

b) Shortage of storage space

c) Explosion especially for explosive medicines

d) Others (Mention)……………………………………

10. In your opinion, what do you think are the barriers to proper disposal of unfit pharmaceuticals?
……………………………………………………………………………………………

Annex II: Checklist on procedures of unfit medicines disposal

S/NO	DESCRIPTION OF INDICATOR	YES	NO
1.	Is there a maintained register book for recording unfit medicines? Check: Date of start………………<br><br>Date of last disposal……………………..<br><br>Type of medicines disposed………………		
2.	Is there a copy of application form for past disposal of unfit medicines? (observe)		
3.	Are unfit medicines segregated from the usable medicines? (observe)		
4.	Are unfit medicines labelled properly? ("Not for sale" in red ink).		
5.	Is there a separate area to keep the unfit medicines?		
6.	Presence of adequate security measures to avoid pilferage (e.g. Grilled gate and windows) for the area to store unfit medicines.		
7.	Presence of previous disposal records (certification of destruction document). Check: Date of issue…………<br><br>Number of disposal per year……………………		
8.	Types of unfit pharmaceuticals found in the facility (in pharmacological groups)		
